# Genetic exchange in eukaryotes through horizontal transfer: connected by the mobilome

**DOI:** 10.1186/s13100-018-0112-9

**Published:** 2018-01-31

**Authors:** Gabriel Luz Wallau, Cristina Vieira, Élgion Lúcio Silva Loreto

**Affiliations:** 1 Entomology Department, Aggeu Magalhães Institute, Oswaldo Cruz Foundation, Recife, PE Brazil; 2 Université de Lyon, Université Lyon 1, CNRS, Laboratoire de Biométrie et Biologie Evolutive, UMR5558, F-69622 Villeurbanne, France; 3 Department of Biochemistry and Molecular Biology, Federal University of Santa Maria, Santa Maria, RS Brazil

**Keywords:** Horizontal transfer, Horizontal transmission, Detection methods, Impact on host genomes, Transposable elements

## Abstract

**Background:**

All living species contain genetic information that was once shared by their common ancestor. DNA is being inherited through generations by vertical transmission (VT) from parents to offspring and from ancestor to descendant species. This process was considered the sole pathway by which biological entities exchange inheritable information. However, Horizontal Transfer (HT), the exchange of genetic information by other means than parents to offspring, was discovered in prokaryotes along with strong evidence showing that it is a very important process by which prokaryotes acquire new genes.

**Main body:**

For some time now, it has been a scientific consensus that HT events were rare and non-relevant for evolution of eukaryotic species, but there is growing evidence supporting that HT is an important and frequent phenomenon in eukaryotes as well.

**Conclusion:**

Here, we will discuss the latest findings regarding HT among eukaryotes, mainly HT of transposons (HTT), establishing HTT once and for all as an important phenomenon that should be taken into consideration to fully understand eukaryotes genome evolution. In addition, we will discuss the latest development methods to detect such events in a broader scale and highlight the new approaches which should be pursued by researchers to fill the knowledge gaps regarding HTT among eukaryotes.

## Background

Inheritance of genetic information begins with DNA replication in parental lines followed by its transfer to offspring or, from an evolutionary perspective, to descendant species. Such process that rules Mendelian inheritance and evolution is known as vertical transmission or Vertical Transfer (VT). However, there is an alternative pathway for genetic information exchange between individuals and species, a phenomenon called Horizontal Transfer (HT). HT is defined as the transfer of DNA fragments between organisms other than through reproduction.

HT is a common process in several Bacteria and Archaea clades being considered a major force driving gene acquisition and hence adaptive evolution in these organisms [1]. In eukaryotes, a small number of HT events were reported until the 1990’s [2] with strong evidence available primarily for the transfer of genomic entities known as transposable elements (TEs) [3]. TEs are selfish mobile genes, capable of using the host molecular machinery for their own replication with no direct benefit to the host genome [4]**.** HT can only occur when a DNA piece successfully invades the genome of the receptor species and is then transmitted by VT to the next generation [5]. Therefore, such events are highly impacted by the different cell/tissue structures, the reproductive mode as well as the degree of interaction of the species involved [6–9]. Several multicellular eukaryotic species present several barriers to DNA exchange by HT such as: (i) cellular differentiation into gametic and somatic cells; (ii) a much lower proportion of gametic cell compared to somatic ones; and (iii) different levels of tissues differentiation.

TEs are commonly known as genomic parasites and, as such, are mostly detrimental to the host [4]. On the other hand, its inherent capacity of mobilization, excision from one DNA loci and insertion into another, increases its chance of invading new genomes compared to non-mobile genes [10].

Since the first HTT reports by Daniels et al. 1984,1990 [3, 11], many other studies were published in which these events were reported in a wide variety of eukaryotic taxa including insects, reptiles, mammals, plants and also between both close and distant related species [12–24]. Up to now, more than 2800 HTT events have been described. HTT between eukaryotes has been drawing a considerable attention in the TE scientific literature for the last two decades that a particular acronym was coined to emphasize its importance: Horizontal Transposon Transfer or Horizontal Transfer of Transposable elements (HTT) [5, 10]. Moreover, a database was created to keep track of new events reported in the literature [25].

In this review we will discuss the latest findings, new methodologies for HTT detection and open questions regarding HTT phenomenon.

## Main text

### New evidence of widespread occurrence

The first described HTT event in eukaryotic species was characterized between species from the *Drosophila* genus and subsequently the majority of new cases (more than 240) were reported between species of this same taxa or other insect species [25–29]**.** The high frequency of HTT in the *Drosophila* genus may be due to a propensity of TEs to invade *Drosophila* genomes by HTT more frequently than other taxa or due to other biological and historical issues [26]. On the one hand, most TEs in *Drosophila* are young and active, being responsible for approximately 80% of all mutation characterized in *Drosophila* [30]**,** overall increasing the chance of an active TE successfully invading a new genome through HTT [12]. On the other hand, there exists a clear bias in the research concerning detection of HTT events, essentially because it is the most known model organism for genetic research, together with the fact that the first HTT event was reported in this genus**.** Several new evidence of HTT among other taxa supports that HTT in *Drosophila* is due to a historical research bias. Below, we discuss new data and extrapolations regarding the HTT extent and frequency based on recent findings.

### HTT in plants

Very few HTT events were reported for some taxa, such as plants and fungi, during the last years. Although some effort had been made to detect HTT events in plants, only 13 HTT cases were reported until 2014 [25, 31–34], maybe because genetic/genomic information was available only for a handful of economically major crop species**.** Since, a significant amount of new plant draft genomes have become available covering most of the Viridiplantae (land plants and green algae) diversity. Exploiting this new data, 3 extensive studies increased the number of HTT events by more than threefold, reaching 50 cases at the time of writing this review. Specifically, **Baidouri and collaborators** [35] performed the most comprehensive HTT study in plant taxa so far, analyzing 40 plant genomes representing the major plant families. This work focused on LTR retrotransposons, the most diverse and abundant TE subclass present in plants genomes, and found 32 new HTT events: 23 events between species from different genus and 9 events between species of different orders. At first, such number does not seem to indicate that HTTs are frequent among plant species when compared to the total number of HTT events reported in Metazoans (2770 events). However, it is important to note the authors only reported HTT events which occurred in the last 3 Mya, besides studied species diverged as much as 149 Mya (CI 148–173 Mya - http://www.timetree.org/) and that DNA transposons were not considered in their analysis. Even though, based on the total number of estimated monocot and eudicot species (13,551), the authors estimated that around 2 million HTT events could have taken place within a 3 Mya time frame. However, it is important to remain cautious with such extrapolations as we know that several host and TE features, not taken into account on these estimates, do influence HTT rate [6].

### HTT in birds

Most of the HTT cases reported so far were described between animal species making up 2772 HTT cases [25]. However, few or no HTT events are known for some taxa, such as birds and unicellular Eukaryotes. This gap has been partially filled with recent findings reporting the first HTT events of a retrotransposon (AviRTE) involving bird species from different orders (Psittacidae and Tinamidae) and parasitic nematodes which nowadays are responsible for several debilitating human diseases such as filariasis and loiasis (*Brugia malayi*, *Wuchereria bancrofti* and *Loa loa*) [36]. TEs consensus sequence reconstructed from each bird and nematode genomes showed a low nucleotide distance of 0.101 substitutions per site which is not compatible with the split time of those species around 758 Mya (CI: 678–916 Mya - http://www.timetree.org/) [37]. Supportive evidence for HTT was also obtained from the TE dating analysis within each genome. A very similar intragenomic TE dating was found in parasitic nematode and bird genomes: i) 25/23 Mya in the ancestor of *Brugia spp.* and *Wuchereria bancrofti* and 4 bird species belonging to *Psittacidae*, *Bucerotidae*, *Trochilidae* and *Tinamidae* bird orders; ii) 22.2/17.7 Mya in the ancestor of *Loa loa* and other 3 bird species which belong to *Suboscines*, *Trogonidae* and *Mesitornithidae* orders. AviRTE elements have a patchy distribution on the avian tree, being found in bird orders that diverged from 79 Mya (Bucerotidae and Trogonidae) up to 110 Mya (Tinamidae and Bucerotidae orders). Such data suggests 2 retrotransposon HT waves between birds from different orders and filarial nematodes occurred: one in the Oligocene period (25 and 23.6 Mya) and a second during the Miocene period (20.2 to 17.7 Mya).

Additionally to this unprecedented finding, the authors made paleogeographic inferences about HTT events based on the bird species harboring the AviRTE element and the pantropical distribution of filarial nematodes. They suggested that AviRTE probably emerged in the neotropical region, since bird species involved in the first HTT events occurred across all tropical regions, except for Madagascar. In addition, the second wave includes species which evolved in Madagascar and took place mainly in the Neotropics suggesting that AviRTE transfers occurred on a global, pantropical scale. These findings support an alternative/speculative view about the origin of filarial nematodes. Current theories suggest that parasitic nematodes emerged in the ancestral mammalian hosts [38]. But, in the light of AviRTE HTT, the origin of these parasites might have happened in the common ancestor of birds, which allowed the transfer of the AviRTE retrotransposons between nematodes and birds, and then infected mammals ancestors [17].

Another recent finding about HTT in birds was the first example of HTT between bird species. Bertocchi et al. 2017 reported the first HTT event of *mariner gallohop* elements between two bird orders: Galliformes and Buceritiformes which diverged around 85–98 Mya [39].

### HTT in insects

The class Insecta represents one of the major eukaryotic evolutionary branches, hence one of the largest species diversity on earth. Several HTTs events have been characterized in insects [40–48], including the first and most well-known case **(refer next topic below)**. Yet, an extensive analysis including most insect orders was still unavailable until recently. Peccoud and collaborators published a study which closed major gaps regarding HTT occurrence in insects. They found at least 2248 HTT occurring events among the 195 insect species analyzed [49]. Due to the extensive amount of data about HTT events as well as the number of species analyzed, they were able to statistically measure some long-standing questions:

i) Does TEs transfer horizontally more frequently in close related species than in more distant related ones?

Yes, they found a significant negative correlation between the number of HTT events and divergence estimates, that is, close related species share more TEs through HT than more distant related species.

ii) Do species which present overlapping habitats share more TEs by HTT than species which are distributed in different environment?

Yes, they found that species which originate from the same region share more TEs by HTT than species which originate in different areas. They also detected a more intense signal for recent transfer than older ones in species that currently share the same area.

iii) Which TEs super families transfers horizontally more frequently?

In agreement with previous findings [5, 25, 50–54] the *Tc1-mariner* elements are responsible for the majority of HTTs (1087 events) followed by *Helitrons* (with less than half of *Tc1-mariner* events), LINE/RTEs, *hAT* and LTR/Gypsy elements. Until recently, limited data about HTT had not allowed to distinguish the HTT rate between DNA transposons and LTR retrotransposons, but clearly showed that those two TE types invaded new genomes by HTT more frequently than non-LTR retrotransposons. Several authors suggested that TEs which are closely related to viruses transfer horizontally due to their capacity to produce infectious viral particles (VPs) and/or use VPs from other viruses [45, 55–58]. If this is true, one should expect that viral-like TEs could transfer frequently between related species due to the receptor host cell constraints for VPs infection. Peccoud and collaborators did not analyse HTT between closely related species, but 112 out of all 353 LTR HTT events reported so far occurred between species of the same genus [25], supporting indirectly the link of infectious viral particles in HTT events of LTR retrotransposons. On the other hand, DNA transposons are simple in structure and can produce recombinant active episomes thus increasing the likelihood of successful transportation between host species [59]. Moreover, such small elements can be easily inserted into viral genomes, active circular forms could be packed into VLPs or even be self-transmissible considering that active episomes can be ingested frequently in a predator/prey or parasitism relationship [60–64]. Altogether, this new data shows that DNA transposons (mainly *Tc1-mariner* and *Helitron*) are tuned for both short and long range HTT events and that viral-like TEs do transfers more frequently among close related species probably due to infective constraints imposed by cell receptor hosts species.

Such large dataset should also allow further insight when ecological data becomes available for the analyzed species such as ecological relationships and their food chain position in the food web. It could help us to decipher the most important ecological factors which impact HTT frequency and distribution.

For a broader discussion on HT in invertebrates refer to Drezen et al. 2016 [9].

### *P* element in *D. simulans*

The *P* element history is the classic example to describe HT among eukaryotic species. It was the first described HTT case reported by Daniel et al. 1984,1990 [3, 11, 65, 66] occurring from *D. willistoni* to *D. melanogaster* species, although several other *P* element HTT were described afterwards [26, 67–70]. This seminal event was particular not only for its unprecedented finding but also for its unquestionable HTT evidences: I) *D. willistoni* and related species presented several copies of the *P* element while *D. melanogaster* was the single species of the melanogaster group to have *P* element in its genome; II) The *D. willistoni* and *D. melanogaster P* element presented only one nucleotide difference although the two species were separated by 54 Mya; III) *P* element intragenomic variability among *D. melanogaster* copies was very low or absent suggesting that it was a very young component of this genome, while in *D. willistoni* genome and related species a higher intragenomic variability could be observed which is compatible with being an ancient genomic component; and IV) all *D. melanogaster* strains collected before 1950 did not contain any trace of the *P* element, while strains collected after 1950 presented the canonical *P* element, with a single mutation differing from *D. willistoni P* element [3].

One unexpected consequence of such transfer was the hybrid dysgenesis syndrome observed when crossing females devoid of *P* element with males containing *P* element [71]. Such cross generated infertile descendants or no offspring at all. Piwi-interacting RNA (piRNA), the small interfering RNAs which regulates TEs at the transcriptional level, is involved in this phenomenon. piRNAs are maternally deposited in the F1 embryos and target TE transcripts [72]. Females lacking *P* elements will not load piRNA in the F1 embryos and therefore high transposition of *P* element affect *Drosophila* development [73].

This is a fascinating and clear-cut example of an HTT event which can have drastic consequences to the host. Now, recent findings are adding new chapters in the *P* element evolutionary history and HTT phenomenon. A new *P* element HTT event was detected occurring between *D. melanogaster* and *D. simulans,* two sister species which diverged from each other around 4 Mya (CI - 2.7 - 9.1 Mya). Kofler and collaborators provided evidence that the *P element* invaded *D. simulans* coming from *D. melanogaster* through a single horizontal transfer event [74]. These authors sampled *D. simulans* populations from South Africa and Florida and performed Pool-Seq sequencing which allowed to measure insertion polymorphism in each population. They found different phases of *P* element invasion: South Africa populations presented 29 *P* element insertions and Florida presented only 4 insertions. The former population was invaded first and is currently in a more advanced phase of invasion and the latter was invaded afterwards, currently being at an early stage of invasion. Moreover, *P* element copies from Florida population were found to segregate at low allele frequencies, which corroborates a recent invasion through HTT. Using a broader *D. simulans* population samples, no *P* element insertions could be found in African (Sub-Saharan) populations collected in 2001/2009 as well as more diverse strain samples from California, North America, Madagascar, New Caledonia and Kenya. The authors found that *D. simulans P* element diverged from *D. melanogaster P* element by only one nucleotide and from *D. willistoni P* element by two nucleotide changes, supporting that the transfer occurred from *D. melanogaster* to *D. simulans*. They also found this same *P* allele segregating at a low frequency in *D. melanogaster* populations suggesting that only one HTT event probably took place, otherwise different *P* elements alleles would be present in *D. simulans* genomes. A hybrid dysgenesis-like syndrome was also found occurring in around 30% of *D. simulans* populations sampled. This subsequent study characterized the HT invasion temporally and geographically surveying 631 *D. simulans* strains collected on three continents covering a time span of 27 years. The authors suggested that *P-*element invasion occurred rapidly since *P* containing strains were rare in 2006 and common in 2014. Moreover, strains collected in the last sampled years presented some degree of resistance to the hybrid dysgenesis phenotype, probably suppressing the *P* element transposition’s deleterious effects [75].

Despite the facts presented for this new *P* element HTT event, the authors concluded that HTT currently only took place in *D. simulans* because of the rarity of an HTT event. Although it can be true in certain situations (on a small scale as months and years), such well characterized events allow us to speculate about HT rates on a broader scale. If we take into consideration that one successful *P element* HT event occurs every ~ 60 years (first between *D. willistoni* - > *D. melanogaster* around 1950*,* second between *D. melanogaster* - > *D. simulans* after 2009), we can expect around 16,000 successful HT cases in 1 My between every two *Drosophila* species. We could add a new layer to this picture considering that there are more than 1700 described species in the *Drosophila* genus [76] alone. Moreover, TEs from other superfamilies, for instance *Tc1-mariner* superfamily elements, transfer horizontally much more frequently, among a broad set of taxa, than elements from the *P* superfamily which are mostly restricted to *Drosophila* genus [25, 52, 77, 78]. However, we should be mindful of the limitation of such estimates given that several host and TE features known to influence HTT rates are not being taken into consideration.

### New evidence for long standing hypothesis: HTT vectors

“Whichever ones are the HTT vectors?” “How and what way does a DNA leaves one organism genome and invade a new one?”. These are long standing questions in the HT field. Until recently, only speculative hypothesis and indirect evidence were available for HTT vectors. In the last couple of years, new technologies have emerged allowing us to perform large scale analysis, hence catching such events. Several host and parasite features have been thought to influence the probability of HTT, including the presence of generalist parasites which can infect more than one host species or symbiotic associations that could mediate TE transfers from one species to another [6].

The first direct evidence of HT possibly mediated by parasites was found under laboratory conditions by Houck and collaborators [79]. *Drosophila* mites (*Proctolaelaps regalis*) fed on eggs of a *D. melanogaster* strain bearing several copies of the *P* element. They later detected that, using several different available methods that *P* element sequences could be carried by the mite, identifying it as the most probable vector responsible for mediating the *P* element HTT event from *D. willistoni* to *D. melanogaster* [79].

Since the 1990’s the TE research community has evaluated other possible vectors with little or no success. However, strong evidence of another HTT vector appeared in a 2010 study from Gilbert and collaborators [80]. The authors found that the triatomine bug *Rhodnius prolixus*, an arthropod parasite of several vertebrate species, presented transposons with more than 98% of identity with opossum and squirrel monkey species, corroborating the hypothesis that parasites can mediate TE transfers among species.

Viruses are other potential HTT vectors identified by several authors due to a number of suggestive features: they can infect a variety of taxa; some have a host genome integration stage; all viruses have an intracellular “life cycle” stage giving opportunity for TEs DNA/RNA packing during viral particles formation and their release after a new viral infection; and some viruses present a gametic cell tropism during infection - an essential step in HTT phenomenon which allows the TE integration into gametic cells and future transmission to the host progeny by VT [81, 82]. Moreover, a clear evolutionary link exists between retrotransposons and retroviruses suggesting that the first still can produce active viral particles [55, 83–87].

Despite all these features, there was only indirect evidence available that viruses could serve as TE vectors between species. However, a single most convincing evidence showing viral genomes containing TE from the host species was reported by Gilbert and collaborators 2014 [88], giving credibility to the virus-TE vector hypothesis. The authors detected two transposons (cut and paste DNA transposons) from cabbage looper transposed to the baculovirus genome *Autographa californica multiple nucleopolyhedrovirus* (AcMNPV) during caterpillar infection and recently demonstrated that those TEs invaded several sympatric moth species by HTT. Using the in vivo experiments associated with high throughput sequencing, they detected that one moth TE transposed into the baculovirus genome every ~ 8500 AcMNPV genomes produced. Caterpillars orally infected with AcMNPV dosage that induced 50% mortality presented tens of thousands of proteinaceous complexes known as occlusion bodies (OB) that allow baculoviruses to remain viable for several years in the environment. A single OB contains ~ 100 virions, each one with several AcMNPV genomes. Hence, caterpillars are infected in the wild with several thousands AcMNPV genomes drastically increasing the change of AcMNPVs genomes incorporating TEs from the host. Moreover, baculoviruses can mount systemic infection in their hosts, infecting several tissues including reproductive cells. There is also evidence showing that individuals from some moth species can survive a high viremic baculovirus infection [89], creating opportunity for TE integration into gametic cells DNA and their passage to the next generations through VT.

In a subsequent study, the same research group identified a continuum influx of moth genetic material in several AcMNPV genomes, showing that not only TEs but also moth genes can be found inserted in viral genomes [90]. These results together with of HTT evidences for several TEs between moth species point towards a very likely HTT vector: the baculoviruses [81]. Another example of viruses mediating HT came from the bracoviruses which were found mediating transfer of bona fide parasitoid wasp genes to the lepidopteran host genomes [91].

Although other vectors still remain a matter of speculations, several authors have highlighted the possibility that phages, endosymbiotic bacteria as well as a wide diversity of parasitic arthropods, mammals and plant species could mediate HTT. Accumulated evidence is showing that endosymbiotic bacteria from the *Wolbachia* genus are likely HTT vectors, because the bacteria genome can integrate in the arthropod partner genome, as exemplified by the *Drosophila ananassae*, *Aedes aegypti* and the pillbug *Armadillidium vulgare* [92–94], and allowing then any carrying TE to transpose into the arthropod genome. As omics technologies continue to advance, unbiased sampling of earth biodiversity will be available to evaluate such open questions. However, at this time, only one study had an experimental design set for evaluation of a likely HTT vector with well postulated null and alternative hypothesis. This study evaluated the role of *Drosophila* parasitoid wasps as HTT vectors using high throughput sequencing in two sets of parasitoid wasps and associated *Drosophila* species in which the parasitoid wasps deposited their eggs [95]. The results showed that parasitoid wasps, at least in the species pairs evaluated, are not an HTT vector. All other studies so far are descriptive in nature and hence did not touch the core questions such as “How and which ecological characteristics influence HTT events”.

### HTT between plants and animals

As data is showing, wherever we compare genomes in any taxonomic level, one can find evidence of HTT (sections above) [96]. Large-scale studies (hundreds of insect species genomes) confirmed the previously suggested hypothesis [10] that close related taxa exchange TEs by horizontal transfer more frequently than divergent ones [49]. Such findings have a major implication on HTT pattern: most HTTs will continue to be found in close related species and we should expect fewer HTT cases in highly divergent species. However, evidence already exist for trans kingdom transfer of transposable elements: Lin et al. 2016 [19] described an ancient horizontal transfer (340Mya) of a *Penelope* retrotransposon from animals to plants (present in conifers but absent in other gymnosperms species) using an array of in silico and molecular techniques. More recently, Gao et al. 2017 [97] showed another evidence of HTT, now of a non-LTR retrotransposon, probably occurring between ancestral aphid or arthropod species to ancestral angiosperms.

### Methods and tools for HTT detection

Historically, HTT have been detected using different types of evidence such as: patchy distribution, higher similarity of TEs sequences when compared with host genes associated with biological information of host species distribution and HTT dating [6]. However, given the increasing pace at which new genomes are being sequenced, a huge analysis bottleneck now exists in all areas of biological studies, including TE studies. Specifically regarding HTT detection, no custom made software was available and researchers were left on their own to implement entire analysis *pipelines* based solely on method descriptions from original papers [98]. Variations or difficulties in implementing those methods could lead to systematic errors as well as analysis repeatability issues. In the face of such challenges, new standardized methods and software have emerged in recent years specifically intended to detect HTT events on larger scale analysis. Below, we will address the benefits and weaknesses of each method.

#### Knowledge-based

One of the most used methods for HTT detection is based on the comparison of genes and TEs distance [26]. Assuming that single copy orthologous genes are vertically inherited, one can estimate nucleotide distance between pairs of host genes and compare this estimates with the TE nucleotide distance found in the same species. If the TE is being inherited by vertical transfer it is expected to have a similar or higher distance than vertically transmitted genes since both TE and genes had the same time to diverge since host taxa speciation. Otherwise, if TEs have significantly lower distance than orthologous genes, it is an evidence that can only be explained by an HTT event [99]. Overall, nucleotide distance analysis has some drawbacks since several evolutionary processes can influence distance estimates as selection pressure on protein level (negative and positive selection). Based on that, Silva and Kidwell 2000 [43, 100] proposed the use of neutral or nearly neutral nucleotides changes, that is, synonymous substitution (dS or kS) changes that do not modify the corresponding amino acid due to the genetic code degeneracy. However, codon bias posed a further issue in that the stronger the codon bias (purifying selection acting at the mRNA level) was, the lower the total number of synonymous changes was in a given gene [101]. Therefore, using genes with strong codons bias deviate the dS distribution of vertically inherited genes to lower values, which can underestimate the number of HTT cases. The most conservative procedure is to estimate codon bias in genes and TEs and only use in comparison genes that present a similar TE codon bias [44, 100]. Although it is a reasonable procedure, it also means that a lot of host species genes/genomic evolutionary information is discarded. Hence, the best approach would be to create a method that accounts for the variation of codon bias and its impact on the dS estimates. With this goal in mind, Wallau and collaborators 2015 [50] developed VHICA (Vertical and Horizontal Transfer Consistency Analysis), an R package with the implementation of a new method accounting for codon bias.

**Fig. 1:**
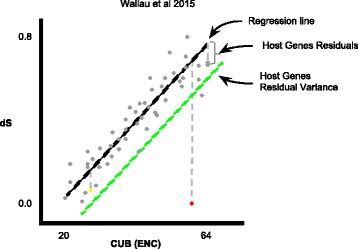
Pairwise regression plot of codon bias and synonymous substitution in which VHICA performs statistical analysis to detected TEs signal departure from host genes. ENC-dS gene estimates (grey circles), TE ENC-dS estimates (red circles), expected regression line (black dotted line), threshold line of *p*-value = 0.05 (green dotted line)

**Fig. 2:**
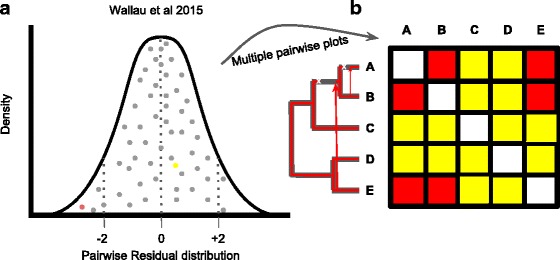
**a** Residual distribution of host genes (grey dots) and TE (yellow - vertically transmitted TE; red dots - horizontally transmitted) ENC-dS from regression line. **b** Graphical representation of several pairwise species comparison and the TE significant departure from host genes distribution (red squares) or not (yellow squares). Red branches represent the TE evolution following vertical transmission among hosts species, white “X” represents the TE lost from the host genome and red arrows represent HTT events

This new method extracts both synonymous substitutions (dS) and codon usage bias (CUB) estimates from codon alignment of vertically transferred host genes and TEs. In a second step, it performs correlation of these values in each species-pair and estimates the expected regression line from the resulting correlation **(**Fig. 1**)**. In the third step, it computes the residuals of VT genes to the expected regression line. TEs residuals from the expected regression line are then calculated and compared with reference genes residual distribution **(**Fig. 2a**)**. Horizontal transfer signal is detected if there is a statistically significant deviation from vertically transmitted genes residuals distribution **(**Fig. 2a and b**)**.

VHICA relies on some biological and statistical assumptions that should be checked before analysis as: Biological - i) reference genes are transmitted vertically; ii) molecular evolution of genes and TEs are similar; iii) horizontal transfer can be interpreted as parsimonious; iv) average CUB between species follow the average evolutionary selection pressure; Statistical – i) relationship between synonymous substitutions and codon bias is linear; ii) residual of the linear regression follows a Gaussian distribution.

All assumptions were validated based on 100 single copy orthologous genes and resampling analysis in the original publication. Specificity and sensibility of the method were validated with several well-known TE HT events among *Drosophila* species (including the famous *P* element case and several other known events from other TEs superfamilies) and have shown to be as much as or more effective than genome-wide methods. Moreover, it was used to characterize several new HTT cases of elements from the *mariner* family of transposons among *Drosophila* genomes.

The implementation of this method in an R package brought the first reusable and standardized procedure to evaluate HT events on a large scale. Other advantages are that this method takes into account the gene and TE codon bias using it as a predictive measure of dS evolution allowing the user to keep all host genes data. In addition, using the R environment, this was implemented with an innovative visualization of both vertically and horizontally transmitted signals taking into consideration the phylogenetic relationship of the host species. Such new visualization, although only qualitative, gives a broad view of the TE family evolution and can guide the detection of HTT directions as well as the discrimination of recent and ancient HTTs.

Drawbacks for this method are to be noted as: i) it relies on coding TEs copies in order to estimate dS and codon bias hence does not allow the HTT detection of elements which lacks coding region; ii) it relies on several assumptions that needs to be checked each time a different set of host species is being analyzed, although literature reports shows that the biological assumptions are well conserved until the family level of different host taxa; iii) A R package allows the generation of figures which can help in the interpretation of the results, but further developments are needed in order to compute a minimum number of HTT events and parsimoniously propose the most likely evolutionary scenario based on the HTT signal detected.

VHICA can be used to analyze several hundred genomes, however, it is important to point out two practical limitation for such analysis: I - VHICA is not a completely functional pipeline in the sense that one need to provide a curated TE and gene datasets input, that is, a multiple codon alignment for each TE family and gene. The challenge here is that codon alignment is not a trivial task especially when considering TEs which are predominantly found fragmented and degraded in the genomes demanding manual curation and/or reconstruction of potential coding region from the most complete copy; II - VHICA was built to analyze each TE family separately, which could be, a time consuming task when TEs from several genomes are analyzed, nevertheless, it is relatively easy to make a loop in the VHICA function to analyze and print the output for all TEs at once.

#### Ab initio

**Fig. 3:**
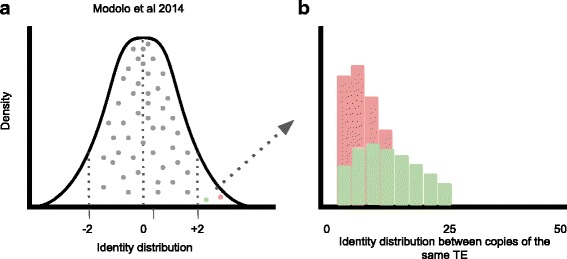
**a** Pairwise genome-wide nucleotide identity distribution extracted from non-overlapping 1 kb windows (grey dots). **b** Second filtering step of putative HTT events, true HTT should presents several highly similar copies corresponding to a transposition burst (red circle and histogram). Green dot and histogram represent a potential HTT event filtered out from further analysis

A new ab initio framework for the detection of HTT events was proposed by Modolo et al. 2014 [102] focusing on a genome-wide detection of all putative horizontal transferred sequences with no prior knowledge regarding TE sequence evolution. Such method is based on an identity approach between two genomes, defining HTT events as a pair of sequences with a higher pairwise nucleotide identity than expected by chance between the two species, and address the detection of all HTTs as a multiple-testing problem in order to control false positive in the results **(**Fig. 3a**).** Further, it implements two validation procedures to control confounding factors: comparative analysis with other species of the phylogeny in order to validate HTs for non-repetitive components of the genome and the use of TEs amplification dynamics expected after an invasion by HTT in a new genome (presence of activity burst = positive HTT) **(**Fig. 3b**).** The authors validated the method with *D. melanogaster* and *D. simulans* genomes detecting 10 new potential HTT events in addition to all known HTT cases already reported in the literature showing the high sensitivity and specificity of this new method.

This new framework overcomes some initial problems related to the previously used methods for HTT detection such as: I) eliminates the bias of analyzing only coding TE sequences or that the coding sequence could be reconstructed; II) can detect TEs and host genes HTs as well; and III) does not require any prior identification of single copy orthologous genes in order to build the H_0_ hypothesis of vertically transferred genes.

This new framework presents tangible advantages, but it also presents some drawbacks: I) although the authors reported some scripts for blast sorting and analysis, there is no custom made package available to detect HTT so far, posing a real challenge for method diffusion and results reproducibility; II) the “Filtering for True Putative HT events” step, which identifies elements with transposition burst signal (several highly similar copies) for further HT analysis, can miss ancient HTT events that have experienced transposition burst but had enough time to accumulate a considerable amount of divergence and hence are excluded as a potential HTT event; III) recently transferred TEs that do not have enough time to accumulate copies could go undetected under this filter as well; IV) up to now, only pairwise genome comparison could be performed without taking into consideration HTT events that probably took place in the ancestral of the analyzed species; and IV) no graphical output is available for the interpretation of HTT events if three or more species are studied.

#### Ecological networks

**Fig. 4:**
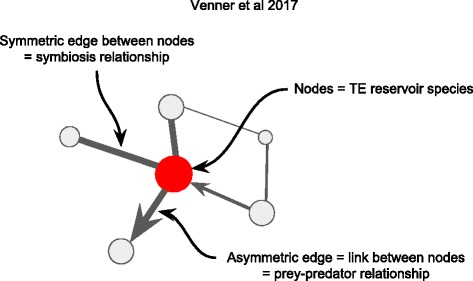
Ecological networks proposed by Venner et al. 2017 which can capture the relationship complexity between species (nodes) and its connections by HTT (edges). Nodes and edges attributes can take into account the importance of a species as HTT hub, the particular presence of HTT catalyzers as parasites (arthropod and viruses), the intensity and directionality of HTT events as well as the type of ecological relationships existing between species

A network-based framework has been proposed recently which could, in principle, integrate more information layers to have a clearer picture of main HTT routes and disentangle the underlying factors behind such phenomenon [103]. Venner and collaborators suggested the use of ecological networks switching from the “*species centric view*” normally used in the HTT detection to an “ecological view” formalized as a network **(**Fig. 4**)**. The sharing of TEs acquired by HTT is the emergent property of the network which can in turn allow the detection of which organism and which of their interactions are prone to promote HTT. “HTT networks”, so called by the authors, have three defining characteristics: i) network topology which defines the organism diversity as well as their functional roles and links; ii) the direction flow within the network which is based on propagation of TEs between species; and iii) the emerging properties of the network **(**Figure 4**).** Although this new framework has not been evaluated with real cases, the authors showed that one can consistently reconstruct those networks based on simulated data. However, several developments as code implementation and validation should be made in the future in order to allow researchers to use it. Moreover, it is important to highlight that such conceptual framework is not a HTT detection method per se but in fact is a way to integrate HTT events detected by the previously discussed methods with ecological features to identify the influence of each feature and species importance in the HTT phenomenon.

Integration of the previous methods, which presented both advantage and disadvantages, in a community driven software package along with further developments would allow a step forward to ensure methods benchmarking, reproducibility, as well as a broad software difusion.

### Further HTT characterization

#### HTT direction ascertaining and dating

HTT direction can be performed in two different ways: based on the parsimony principle or the TE age.

The parsimony principle was frequently used in the first publications of HTT events since elements ages were difficult to access without full genome assembly. Such principle is based on the reasoning that, if a given taxon has a higher amount of species hosting a particular TE in comparison with another taxon where only some species bears it, most probably the first is the donor and the last is the receptor taxa [104].

TEs elements ages inside each genome can be estimated applying molecular clock using an estimated evolutionary rate. A consensus copy is reconstructed which represents the ancestral element and the distance of each current copy is estimated against the consensus copy. Then, using the evolutionary rate, the elapsed time can be estimated for all copies establishing a lower bound date for the entrance of the TE in the receptor genome. Most of the time there is no TE specific evolutionary rate available, but with the availability of whole genomes one can estimate it from ortholog copies found in different genomes (as far as there is available dating for host speciation) [15, 105]. Another strategy used to estimate intragenomic elements age is based on the distance of two long terminal repeats (LTRs) of retrotransposons. Such estimates are based on the reasoning that LTRs start to accumulate mutation independently as soon as a new copy is inserted into a new loci, hence estimating the distance between the two LTRs and using a specific molecular rate one can estimate the elapsed time of each copy in the genome [106]. With this data we can infer in which species the elements are older or younger and indicate the transfer from the older to the younger containing TE species.

#### Complementary evidence

Although currently the data about HTT is more comprehensive and detection methods more sophisticated, it is important to highlight that any complementary evidence which supports HTT is highly valuable. For instance, besides obtaining significant *p*-values for HTT events with the software mentioned earlier, the detection of patchy distribution and phylogenetic incongruences of host and TEs phylogenetic trees, which can corroborate these events, is recommended. Moreover, plausible hypotheses about the ecological characteristics which allowed HTT to happen, such as overlapping habitat or sharing of parasites by the HTT involved species or overlapping habitats of ancestral lineage when ancient HTT are detected, are recommended as soon as this information is available [40, 107, 108].

Another important step in the characterization of HTT events is to verify TE integration into the host receptor genomes, that is, evaluate if TEs with HTT signal may be any sort of contamination or not. As many genomes available are fragmented and different approaches have been used to avoid contamination, any further evidence showing that TE involved in the HTT is an integral part of the species genomes is needed. Two strategies can be taken: I - in silico evidence: the detection of more than one copy in the genome; such copies should be found in the inner portion of contigs or scaffolds surrounded by unique host species sequences (not in their extremities which normally happens for wrongly assembled sequences); raw sequence reads mapped on the specific insertion site since true integration, not derived from assembly errors, are expected to show similar coverage depth than flanking (host species) sequences; II - additional molecular data showing the integration of the element as PCR insertion specific amplification and chromosome mapping with FISH or other hybridization approaches. A more in depth and detailed discussion about validation and challenges in HTT detection on a large scale focusing on in silico analysis can be found at *Peccoud* et al. *2017* [96].

## Conclusions and perspectives

### Future directions and prospects

As expected in any scientific field some answers open up an entire set of new questions about a given phenomenon and HTT phenomenon is no exception. The availability of new genomes allowed us to detect HTT in a wide variety of taxa and we can now devise some raw, yet very speculative, estimates of those events in nature. Moreover, broad scale studies as reported throughout this review allow us to identify TEs families which are prone to undergo horizontal transfer between species, new interacting species which exchange TEs frequently as well as biological and ecological conditions which may influence the occurrence of such transfers. Due to our current knowledge about the mobilome of those host species or communities and the current ‘low cost’ high throughput sequencing, we can now set long term experiments to track HTTs using different approaches as suggested below:

### Species-pair approach

A set of interacting species should be chosen, which match a number of premises thought to enable TE exchange by HTT. By this, we mean an intimate ecological relationship as predator-prey and parasitism relationships along with mobilome features, such as high incidence of HTT events previously identified between those species and a young mobilome with several active TEs which are more likely to transfer between species. Moreover, it would be an advantage if such pair of species could breed in laboratory conditions combined with small generation time and large number of descendants. Such species-pair should evolve mimicking natural conditions: foraging in the same site or sharing of parasites. The mobilome of those species could them be characterized prior to the experiment. Thus, we could precisely and timely determine new HTT events performing new rounds of genome sequencing and mobilome characterization after species interaction. However, it is important to point out that tightly controlled experiments are needed together with several lines of evidence showing that a given TE was transferred horizontally between those species to avoid the possibility of contamination.

Finding such species pairs which match all HTT interesting premises may be difficult but several interacting entities are good candidates. One example is predator-prey relationship which has been shown to influence HTT events between insectivorous bats and insects [21] and impact directly the chance of endosymbiotic bacteria as *Wolbachia* to transfer horizontally from prey to the predator [109, 110]. However, conflicting results were observed from different predator-prey species sets [111, 112]. Such differences are expected due to specificities of each interaction such as: the encounter frequency, the infectivity of the potential vector entity (bacteria lineage) and the ability to invade a new host cell which can have significant molecular differences than the previous host. Another important point is that, based on the features of the predator-prey species pairs, one can design experiments which increase HTT opportunities to take place, that is, depending on the phylogenetic distance between the two involved species. Another example is the virus-host parasitic relationship, since virus genomes do not only exchange DNA with their host genomes but are also identified as one of the main HTT vectors which could mediate the transfer of a given TE to a new host (discussed in section **New evidence for long standing hypothesis: HTT vectors**). Several viral features are compatible with the hypothesis of being a vector for TE transfer as reported before in the **New evidence for long standing hypothesis: HTT vectors** section. Based on that one can set up experiments with viruses which have a wide-host spectra, produce mild infection which can reach germline cells and do not kill most of the infected host individuals and that can integrate themselves into the host genome. An experiment could also be proposed where the viral genome is reconstructed with an active TE insertion and then allow its propagation in a specific host which the TE insertions can be tracked with different molecular techniques. The fact that viruses replicate abundantly will increase the chance of HTT happening.

Symbiosis is another ecological relationship usually highlighted as having great potential for HT opportunity since it is normally the outcome of a long interaction and evolutionary trajectory. Therefore, we have should expect to detect more ancient HTT events and maybe are not well suited for recent HTT detection.

### Community level approach

Such approach is clearly more complex since it is expected to track not one or two host species mobilome but, in fact, several interacting species mobilomes, thus presenting an array of ecological relationships in a community. Therefore, obtaining genome-wide information and mobilome description for each species could be challenging and very expensive even with the decreasing cost of sequencing. In order to plan such an experiment, a target sequencing approach could be fine-tuned to obtain information of just a fraction of the Mobilome: the active mobilome. Some methodologies as mobilome-seq [113] could be used to select only active transposable elements in different organisms which can then be compared from time to time for HTT detection. Another important challenge is how to identify most species from a large set of taxa in order to allow a more fine-grained analysis of HTT events. Such community based strategy would be the ideal approach to evaluate the premise and predictions of “Network Method” discussed above.

Another interesting opportunity to explore HTT phenomenon is to follow invasive species populations in real time during the invasion of a new environment. The Mobilome of such species could be characterized by sequencing the genome of ancestral populations from sites where the species originated, and comparing with genomic information of invasive populations as well as interacting species in the new environment. Invasive species usually have a huge increase in population size after an initial bottleneck and are expected to have a higher rate of exposure to HTT events [114]. Moreover, such species might be more permissive to infection by parasites in a new environment, thus making them particularly permissive to HTT. Therefore, it would be a good opportunity to map new HTT events and understand the impact of a new TE in a genome at the molecular level.

### Unexplored taxa and intriguing questions

Although our knowledge at the molecular level (genome studies) continue to increase rapidly in different eukaryotic tree branches, there are still large bias of HTT reports in multicellular eukaryotic species. As highlighted earlier, one of the main physical barriers of HTT in multicellular eukaryotic species is the infection of the germline. Those cells normally represent a small fraction of the total cells of the organism and are usually surrounded by other tissues. Germline tissues are also characterized by a large arsenal of molecular weapons to regulate and disable parasites (highly active piRNA machinery [115]). However, some still understudied taxa of unicellular free living eukaryotic species or multicellular eukaryotes with few or no tissue differentiation could become a much better model species due to the almost absence of such barriers. Such species are expected to experience a much higher HTT rate but, at the same time, eliminate TEs rapidly as well due to their compact genomes and large population size. Therefore, the understanding of the HTT phenomenon could benefit from studies focusing on eukaryotic less differentiated organisms or with absence of tissue separation of soma and germline. Some lines of evidence are already pointing to this direction. For instance, the planaria and hydra species were subject to several HTT events [116, 117].

Another still understudied taxa regarding HTT is fungi. So far only 9 HTT cases have been well characterized [118–121]. But extensive TE characterization of more diverse set of fungi species suggests other potential HTT events [122]. However, in depth analysis with software developed specifically for HTT detection is needed.

Regarding HTT in plants (refer HTT in plants subsection), several new events were uncovered in a large scale study [35]. However, comparing the number of transfers found between plant species (32 HTTs of retrotransposons - 40 genomes surveyed - 0,8 HTTs normalized by the number of genomes) with the most extensive detection of HTT between animal species (2248 HTTs found, 435 HTTs considering only retrotransposons - 195 genomes surveyed - 11,52 HTTs normalized by the number of genomes, 2,23 considering only retrotransposons) [49] indicate a lower HTT rate in plants. 14,4 orders of magnitude more HTTs in animals considering all TEs studied and 2,4 orders of magnitude more HTTs in animals considering only retrotransposons. Therefore, although it is still difficult to separate the influencing factors, it is interesting to speculate that plants seem to have some barrier to the entrance of genetic parasites like transposable elements through HTT.

Based on the similarity of retrotransposons with viruses, we can suppose that they self-propagate to other species through viral-like infection and evolved mechanisms to target germline cells. However, such strategy can have different efficiencies in plant and animal species. Plant species, mainly flowering plants, do not have a clear distinction between germline and somatic cells, the former being originated from single somatic cell [123, 124]. TEs which invade host species by viral-like infection and target germline cells may be very efficient in invading animal germline but not plant species, which would explain the different rates of HTT between them.

### Somatic transposition and asexual reproduction

Evolutionary studies addressing HTT assume that invasion of germ cells is an essential step for TEs invasion and the spread into the receptor genome while TEs acquisition by somatic cells can affect an organism during its life time, but are irrelevant in the evolutionary context since they will not be inherited by the next generation. However, these assumptions are valid only for organisms showing sexual reproduction and an early separation between germ and somatic cells. Therefore, such assumption ignores an important fraction of eukaryotic species including several invertebrates, unicellular eukaryotes, as well as the majority of plants. All such species lack segregation between germline and somatic cells and/or germ cells can arise several times from independent somatic stem cells [125–127]. In those species, TEs horizontally transferred to somatic tissues can be incorporated into the genome and propagated for the descendants.

**Fig. 5:**
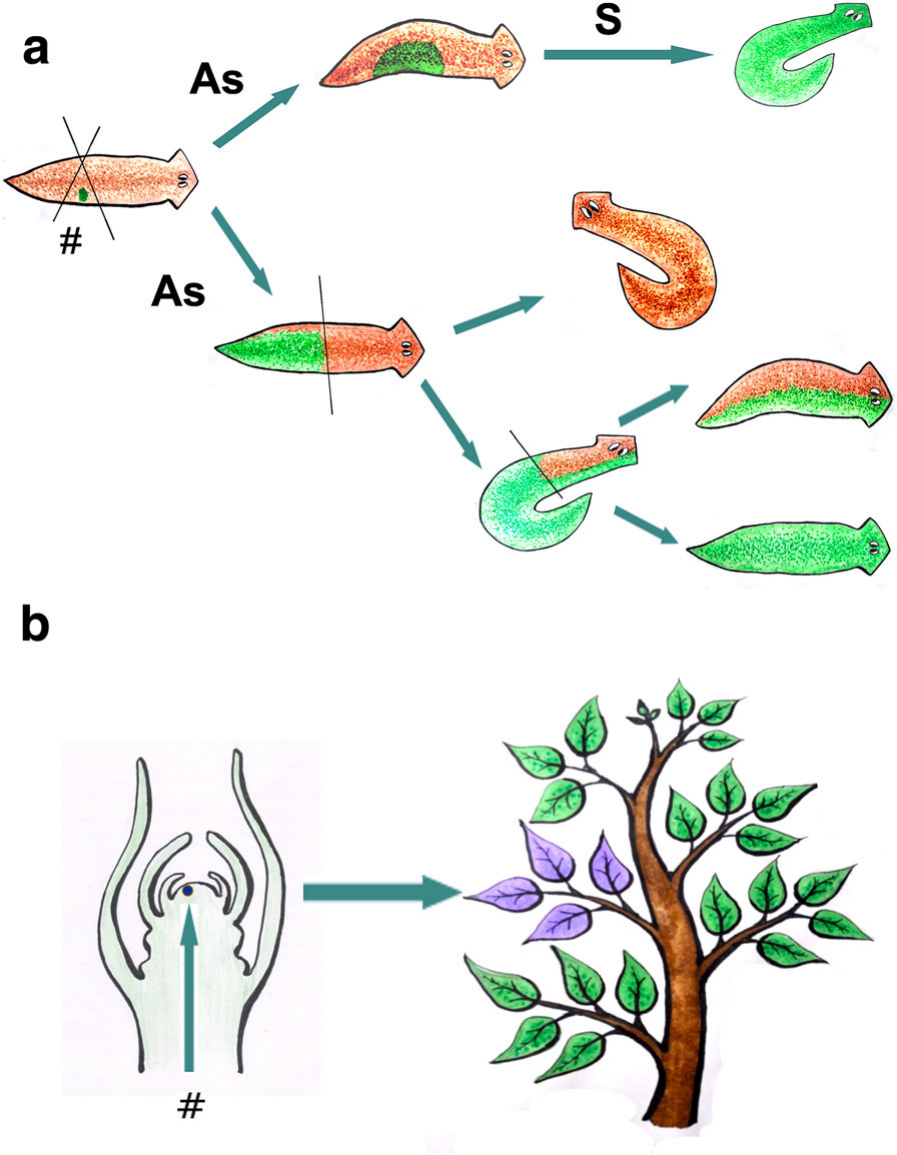
Somatic TE invasion through HTT in eukaryotic organisms with few or no cell differentiation and asexual reproduction. **a** Organisms alternating sexual and asexual phases showing regenerative capacity - somatic TE invasion (# - green spot). Considering that new organisms can emerge by regeneration (asexual reproduction - As) or sexual reproduction (S), when gonads arise from the cell invaded by the TE, the descendants will bear the new horizontally transferred TE in their genome in different proportions. **b** In plants with cycles of sexual and asexual reproduction, a somatic TE invasion of a meristematic cell by horizontal transfer can give rise to descendants containing the TE insertion

Theoretical models show that asexual reproduction can have a different impact on newly arrived TEs in a receptor genome depending mostly on the population effective size of the species. Large populations tend to eliminate TEs, while in small populations TEs may be extinct due to the Muller’s ratchet effect, promoted by their deleterious effect [128]. TE data from asexual organisms, such as the Bdelloid rotifers, strengthen this hypothesis showing few TEs in their genomes which are mostly acquired by HT [129, 130]. In addition, many organisms use both reproductive processes, interchanging sexual and asexual phases. Somatic transposition associated with the alternatives cycles of sexual/asexual reproduction can have a strong influence in the HTT rate in these organisms as well (Fig. 5). The large TE content present in the Hydra genome, which a large portion was acquired by HTT, could be attributed to the somatic “acquisition” and the sexual/asexual reproduction cycles [116].

The distribution of HTT events along the eukaryotic tree should be reevaluated keeping in mind the differences in the reproductive modes observed among eukaryotic species along with the role of somatic invasion by TEs. Somatic TE acquisition may be irrelevant in some reproductive systems but can be important in others. Somatic transposition and reproductive systems need more attention in HTT studies.

### Could HTT be used to make inferences on host biology?

Massive detection of HTT events in taxa can reveal several host-permissive features which allow the exchange of genetic material between species. Information about permissiveness of parasites exchange such as TEs can be a proxy for the likelihood of other parasites (viruses and bacteria) exchange between host species. Several parasites are now being used for biological control of insect pests such as pathogen-vector mosquitoes and knowledge of the probability of parasites escape to other non-target species through HT is important information to consider when evaluating biological control safety.

Such data can also be explored to generate new hypotheses and insights about host biology such as species interaction which occurred in the past allowing the exchange of parasites (refer HTT in birds above and reference [25]) or detection of reservoir species.

In summary, having a more detailed view of HT phenomenon among eukaryotic species can improve our ability to understand host/parasite coevolution as well as exploit this information for development of new biotechnology [96].

### Should we reconsider eukaryotes phylogenetic trees as networks as proposed for prokaryotes?

The rate with which new HTT findings are reported among distant and close related eukaryotic species is showing that representing the evolution of eukaryotes using only tree like structures is not satisfactory and that connection between trees branches other than by vertical transfer should be considered. However, different from Bacteria and Archaea most genes are transmitted vertically and the HT extent in eukaryotes are orders of magnitude lower than in prokaryotes even only considering TEs. Therefore, the best representation of eukaryotes evolutionary relationships should be the traditional tree like structure but with the addition of intermediate link representing HTT events.

### HTT and impact on eukaryotes evolution

Understanding the impact of TEs on host genomes is one of the most active research areas, yet still with few clear demonstrated examples only in model organisms. Most of the HTT events characterized so far were described in non-model species challenging the evaluation of their impact on the receptor genome. Besides, a recently arrived TE usually undergoes a transposition burst, where a TE can reach from tenth to thousands of copies until elements degenerates or the host regulatory machinery begin to regulate it. At this amplification phase, a newly arrived TE is more likely to generate adaptive insertions which brings new advantageous features to the host genome. HTT may have an impact on genome size. For example, Peccoud et al. 2017 estimated that transferred TEs contribute on average 2% of the insect genome but this number could be up to 24% for some species [49]. Other studies showed indirect evidence of HTT and transposition burst shortly after such events associated with polyploidy and speciation events in salmonids [131] and mammals [132, 133]. Another known example was the co-option of a DNA transposons which underwent horizontal transfer and emerged as a fused new gene specific to murine rodent species [15]. Moreover, the most well-known and, as far as we are aware, the single most well-understood case of TE invasion by HT associated with impact on the host genome is the *P* element and hybrid dysgenesis phenotype described in section (“*P* element in *Drosophila simulans”*). Therefore, although it is likely that horizontally transferred TEs do have an impact on host genomes, we still need to experimentally test this in several species. HTT impact and consequences can change the way we understand species involved in HT evolution and eukaryote evolution based on the promiscuity of such events.

### Databasing and data availability

The unprecedented amount of data about HTT is allowing a quantitative assessment of several questions as reported earlier. However, it also poses new challenges such as: I - new findings should be checked carefully against previous findings to remove information duplication; II - in order to make inferences about ecological factors a good databasing is necessary to keep track of all information and connectivity between them; III - the need for benchmarking and performance comparison of the different detection methods; and IV - wide application of standard methods in several taxa.

With those new challenges in mind we call all researchers working with TEs and HTT to make available as much data as possible to incorporate into HTT database, thus ensuring that future studies can use the information to check against previous detected transfer and benchmarking new methods and pipelines.

In conclusion, HTT among eukaryotes can now be seen as a real and important phenomenon for host genome evolution. Moreover, we can consistently affirm that new events will continue to be found following the increased use of new software and approaches specifically designed to explore the evolution of TEs in under explored taxa giving us a better view of the TE network and its impact across Eukaryote evolution.

## Abbreviations

CUB: Codon Usage Bias
dS: Synonymous Substitutions
ENC: Effective Number of Codons
HT: Horizontal Transfer
HTT: Horizontal Transfer of Transposable elements
TE: Transposable Elements
VHICA: Vertical and Horizontal Transfer Consistency Analysis
VT: Vertical Transfer
